# Next‐Generation Strategies for Neural Repair and Regeneration: Neural Organoid Transplantation in the CNS

**DOI:** 10.1111/cpr.70223

**Published:** 2026-05-01

**Authors:** Yutong Wang, Jinrui Li, Xingjia Mao, Wanyu Wang, Yansong Li, Xuefei Hu, Jinjie Zhong, Fengdong Zhao, Linlin Wang

**Affiliations:** ^1^ Department of Orthopaedics of Sir Run Run Shaw Hospital, and Department of Basic Medicine Sciences Zhejiang University School of Medicine Hangzhou Zhejiang China; ^2^ Department of Neurosurgery Second Affiliated Hospital of Zhejiang University School of Medicine Hangzhou China; ^3^ School of Clinical Medicine, the Affiliated Hospital of Hangzhou Normal University Hangzhou Zhejiang China; ^4^ Department of Basic Medicine Sciences Tarim University Aral People's Republic of China; ^5^ Department of Obstetrics of the Second Affiliated Hospital, and Department of Basic Medicine Sciences Zhejiang University School of Medicine Hangzhou Zhejiang China; ^6^ Department of Orthopaedic Surgery, Sir Run Run Shaw Hospital Zhejiang University School of Medicine Hangzhou China

**Keywords:** characteristics, maturation, neural organoids, repair and regeneration, transplantation

## Abstract

Neurological disorders are often devastating and notoriously difficult to repair, creating an urgent need for novel research models and therapeutic strategies. Neural organoids—three‐dimensional, self‐assembling structures derived from stem cells—have emerged as a powerful platform to address this challenge. Supported by enabling technologies like bioreactors and 3D printing, advanced maturation protocols have significantly enhanced their cellular diversity and functional utility. This progress has paved the way for their widespread application in developmental studies, disease modelling, and notably, regenerative medicine. Focusing specifically on the latter, this article reviews how neural organoid transplantation opens new avenues for treating CNS injuries and degeneration. We first elaborate on the development, characteristics, and maturation strategies of neural organoids. We then summarise the translational applications and achievements of transplanting both whole neural organoids and their derived vesicles, analyse the prevailing challenges in the field, and finally, outline future directions to advance the therapeutic potential of this technology.

## Graphical abstract

Neurological disorders are hard to treat. Stem cell‐derived neural organoids enable research, and their transplantation aids CNS therapy, with this article reviewing relevant advances, challenges and prospects.

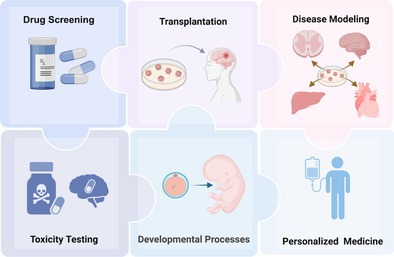

## 1 Introduction

Organoids are three‐dimensional self‐organising structures derived from stem cells that recapitulate the key features of the structure and function of native organs. By optimising the combination of growth factors and refining cell isolation protocols, researchers have successfully generated a variety of organoid types that mimic in vivo organs, including brain [1], tendon [2], and retinal [3, 4] organoids. Furthermore, with the advancement of novel technologies such as bioreactors [5, 6], microfluidics [7, 8, 9], 3D bioprinting [10, 11, 12], and artificial intelligence [13, 14], organoid research has stepped into a high‐quality development stage. Recent studies have demonstrated that brain organoids can spontaneously generate highly structured and strictly ordered neural firing sequences in the complete absence of external input. Such mature brain‐like physiological characteristics highlight the broad prospects of neural organoids for future applications [15].

**FIGURE 1:**
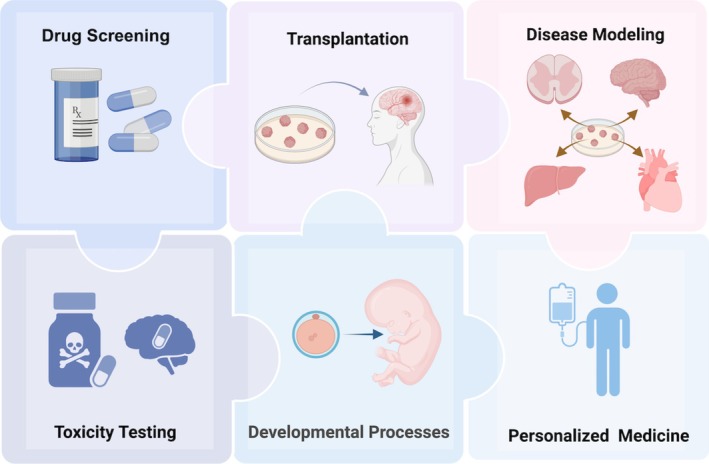
Multiple applications of organoids.

Leveraging their high physiological relevance and controllability, organoids have emerged as versatile tools across diverse research domains (Figure 1). Primarily, they recapitulate key in vivo developmental processes, enabling mechanistic investigations into how factors like genetic regulation and microenvironmental signals shape tissue development. A cornerstone application lies in disease modelling. By faithfully replicating human tissue architecture and cellular complexity in vitro, organoids facilitate the dissection of pathogenic mechanisms [16, 17]. Their similarity to human tissues also makes them invaluable for drug screening and toxicity assessment, serving as a reliable preclinical platform [18, 19].

In recent years, organoids have garnered significant attention for their enormous potential in regenerative medicine. They can self‐organise into functional, three‐dimensional structures mirroring target organs. Upon transplantation, these organoids exhibit rapid integration into host tissues, highlighting their capacity to drive local tissue repair and regeneration.

Today, organoids are indispensable core tools in medical research [20, 21]. Central nervous system (CNS) diseases are among the leading causes of death and permanent disability worldwide, and also represent one of the greatest challenges in current healthcare [22]. Millions of patients suffering from CNS diseases bear the burden of neurological tissue dysfunction [23], such as stroke [24, 25], traumatic brain injury (TBI) [26, 27], spinal cord injury (SCI) [28, 29], Alzheimer's disease (AD) [30], and so on [31, 32, 33]. These diseases are characterised by inaccessibility and high complexity. In traditional research, animal models and post‐mortem brain tissues have been the primary tools for studying CNS diseases. However, significant disparities exist between animal models and the human brain in terms of physiology and anatomical structure. On the other hand, post‐mortem brain tissues raise ethical concerns as well as poorly controlled quality, failing to reflect dynamic neurophysiological activities under in vivo physiological conditions [34, 35].

The limited regenerative capacity of neural tissue is the main reason for human disability and mortality. Currently, varying neurorestorative therapeutic strategies have been tried to improve the daily life quality for patients [36]. Drugs and surgery can alleviate symptoms; however, they fail to achieve fundamental structural and functional repair [37]. Effective intervention may give more benefits for those patients, seeking research platforms that more accurately mimic human biology. Against this backdrop, the rapid advancement of CNS organoids offers a promising alternative, significantly enhancing the understanding of human nervous system development [38, 39] and various neurological disease mechanisms [40] while driving innovation in novel therapeutic approaches [41]. Consequently, it provides new tools and perspectives for fundamental research, disease modelling, drug development, and personalised therapy in neuroscience, elevating the platform for disease investigation and treatment.

Importantly, CNS organoid transplantation can replenish damaged or missing neurons and repair neural circuits, thereby improving neurological function [42]. However, translating this approach from proof‐of‐concept into a reliable therapy requires ensuring long‐term graft survival, precise neural circuit integration, and functional recovery. In this review, we focus on the role of neural organoid transplantation in CNS repair and regeneration. We first introduce the development history of neural organoids and their characteristics, as well as the strategies for enhancing the maturity of neural organoids. Second, we summarise the application and achievements of transplantation by using neural organoids, neural organoid‐derived extracellular vesicles, and exosomes for CNS repair and regeneration. Finally, we further point out the current challenges and indicate the future research directions in this field.

## 2 Development and Characteristics of Neural Organoids

### 2.1 Development of Neural Organoids

**FIGURE 2:**
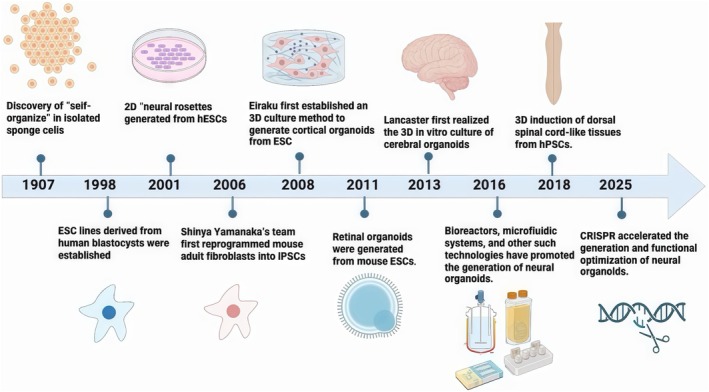
Development history of neural organoids.

As summarised in Figure 2, neural organoids have undergone a progressive developmental process. These 3D in vitro aggregates arise from stem cells, which self‐renew and differentiate under inductive conditions to form hierarchically organised, functional tissues [38, 43]. The earliest description of this in vitro regenerative capacity in cells dates back to 1907, when Wilson [44] discovered in sponge cell cultures that isolated sponge cells could reaggregate and self‐organise into new, fully functional sponge organisms. This marked the first attempt at in vitro biological issue reconstruction. Over the following century, stem cell technology underwent tremendous breakthroughs and innovations. In 1998, the first human embryonic stem cell (hESC) lines were established from human blastocysts [45]. Three years later, exposure to fibroblast growth factor 2 induced hESCs to differentiate into polarised neural epithelia termed neural rosettes, a 2D polarised organisation of neurepithelial cells [46, 47].

However, these systems lacked the brain‐specific spatial structure and morphology and were insufficient in accurately replicating the complex morphology and physiological functions of real tissues. A paradigm shift occurred in 2006, when Takahashi and Yamanaka reprogrammed somatic cells into induced pluripotent stem cells (iPSCs), revolutionising cellular plasticity and infusing new vitality into regenerative medicine [48]. These studies revolutionised the understanding of stem cell plasticity mechanisms and opened up new avenues for regenerative medicine.

The transition to bona fide organoid culture occurred in 2008: Eiraku et al. [49] developed a 3D method to generate self‐organising cortical tissue from ESCs, bridging the gap from 2D to 3D tissue morphogenesis. The landmark achievement, however, came in 2013 when Lancaster encapsulated human pluripotent stem cells (hPSCs) in Matrigel to generate the first cerebral organoids [50]. These structures accurately recapitulated early human neurodevelopment [38], spontaneously patterning into distinct regional identities, such as the forebrain [51, 52], choroid plexus [53, 54], and hippocampus [55].

This 3D paradigm fundamentally addressed limitations of 2D culture by recapitulating the in vivo cellular microenvironment and enhancing cell–cell interactions. Subsequently, diverse neural organoids, such as spinal cord [56] and retinal organoids [57], have emerged, enabling mechanistic studies of nervous system development and offering promising translational avenues for regenerative medicine.

Structurally and morphologically, neural organoids share high analogy with the human cerebral cortex in terms of cellular composition, differentiation pathways, and functional gene expression [58, 59, 60]. Their development recapitulates human foetal brain ontogeny [61], yielding cortical tissues with genetic programs highly reminiscent of foetal tissues [38, 59]. Moreover, brain organoids spontaneously exhibit cortical region‐specific patterning and molecular asymmetry [62, 63], which closely matches the characteristics of the authentic human cerebral cortex. Given the inaccessibility of research samples for psychiatric disorders [64, 65], patient‐induced pluripotent stem cell (iPSC)‐derived neural organoids serve as clinically relevant and valid models, as they can accurately recapitulate the abnormal neurodevelopmental trajectories and core pathological mechanisms of these disorders [66, 67, 68].

**FIGURE 3:**
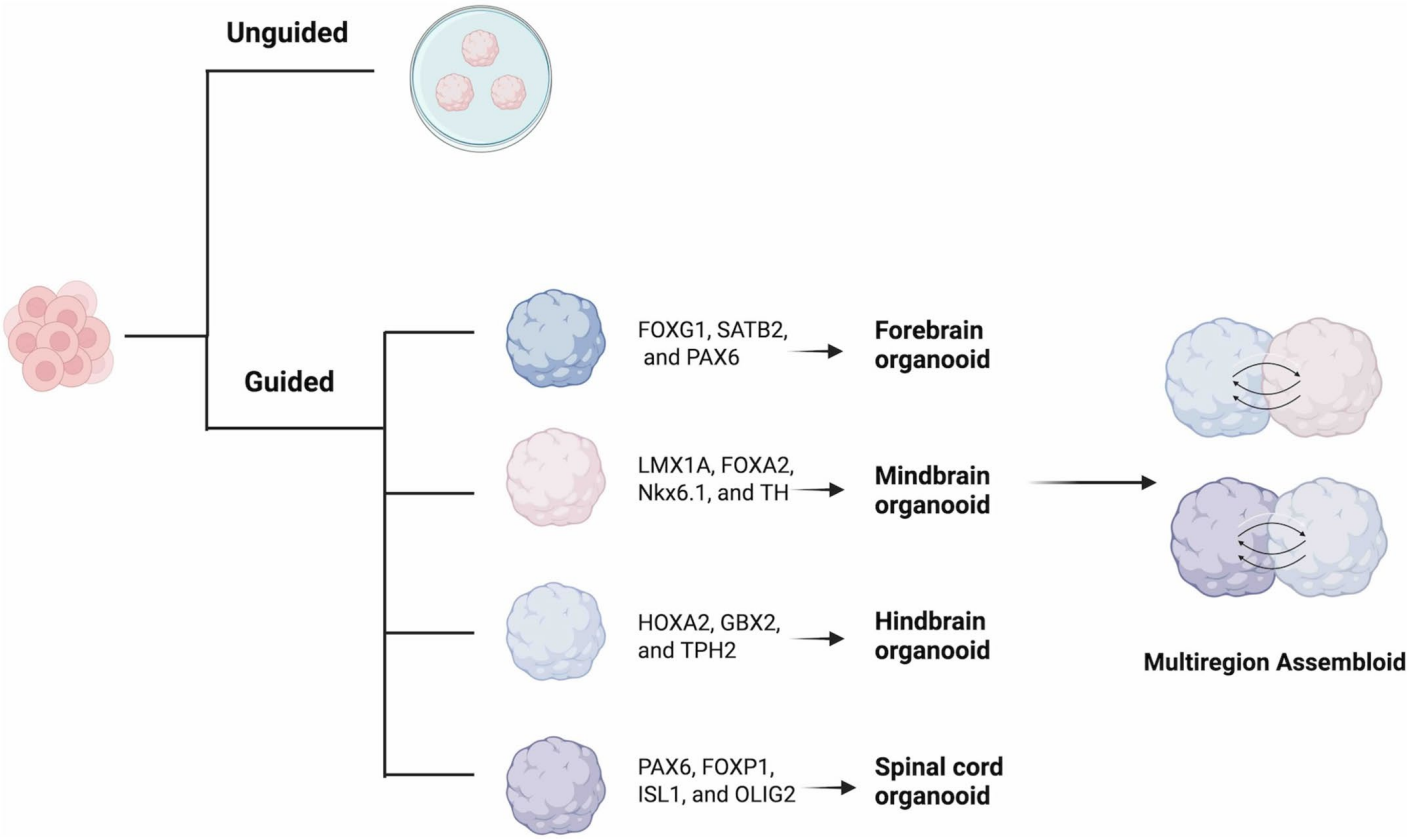
Methods for neural organoid.

Generally, the generation of neuron organoids can be divided into unguided and guided methods (Figure 3). The unguided method allows cells to differentiate spontaneously into heterogeneous tissues and recapitulate brain structures, developing in a manner similar to the authentic human brain [50, 69, 70, 71]. The guided method enables the directed induction of specific brain regions by adding exogenous regions, allowing for the precise generation of brain region‐specific organoids—such as forebrain [72], midbrain [73], thalamic [74], or cerebellar [75] spinal cord organoids [76, 77].

Single‐region brain organoids lack non‐neuroectodermal cell populations, such as microglia and endothelial cells, yet these cell types are essential for brain function. With the advancement of mature culture techniques, numerous studies have focused on expanding single‐region brain organoids to heterogeneous multi‐region brain organoids, which could improve the in vitro simulation of the complex structures and functional connections of the human brain [78, 79]. For example, the team led by Masatoshi Nishimura used hiPSCs to create thalamocortical assembloids, which precisely reconstruct the thalamocortical interactions and capture the early mechanisms of brain development [80]. In the latest research, investigators constructed a human miBrain model comprising six types of CNS cells, exhibiting physiological neural activity and a blood‐brain barrier [81, 82].

### 2.2 Characteristics of Neural Organoid

Region‐specific neuron organoids can be distinguished and identified through their distinct marker molecules (Table 1). For forebrain organoids, the key markers are FOXG1, SATB2, and PAX6 [51, 52]. Midbrain organoids are characterised by markers including LMX1A, FOXA2, Nkx6.1, and TH [85, 86]. Hindbrain organoids express markers HOXA2, GBX2, and TPH2 [1, 88]. Cerebellar organoids are identified by PAX6, CALB1, PTF1A, and MATH1 [90, 91], while cortical organoids are distinguished by EMX1, SLC17A7, and NEUROD6 [94, 95]. Hippocampal organoids use HOPX and PROX1 as markers [97]. Thalamic organoids are characterised by NRP1, ECEL1, and TCF7L2 [98, 99]. Hypothalamic organoids have markers including RAX, OTP, NKX2‐1, and POMC [100, 101]. Striatal organoids are identified by DARPP32 and CTIP2 [102]. Choroid plexus organoids use markers such as TTR, CLIC6, and MSX1 [50]. Pituitary organoids rely on markers including ACTH, TBX19, PRL, and PITX1 [100, 106]. Retinal organoids are distinguished by markers such as CHX10, VSX2, RHO, BRN3A, MITF, CRX, and GS [108, 109]. Finally, spinal cord organoids are characterised by PAX6, FOXP1, ISL1, and OLIG2 [28, 112].

**TABLE 1:** Region‐specific markers of neural organoids.

Region‐specific brain organoids	Markers	Detect tissues or cells	
Forebrain organoid	FOXG1	Telencephalic cells	[51, 52, 83, 84]
	SATB2	Forebrain superficial cortical neurons	
	PAX6	Forebrain neural progenitors	
Midbrain organoids	LMX1A	Dorsal midbrain	[85, 86, 87]
	FOXA2, Nkx6.1	Ventral midbrain	
	TH	Dopaminergic neurons	
Hindbrain	HOXA2	Anteroposterior axis differentiation of the hindbrain	[1, 88, 89]
	GBX2	Hindbrain	
	TPH2	Serotonergic neurons	
Cerebellar organoids	PAX6	External granular layer of the cerebellum	[90, 91, 92, 93]
	CALB1, PTF1A	Cerebellar Purkinje cells	
	MATH1	Cerebellar granule cells	
Cortical organoids	EMX1	Cerebral cortex progenitors	[94, 95, 96]
	SLC17A7	Dorsal cortical excitatory neurons	
	NEUROD6	Immature‐to‐mature excitatory neurons	
Hippocampal organoids	HOPX	Hippocampal progenitors and mature neurons	[97]
	PROX1	Hippocampal dentate gyrus granule cells	
Thalamic organoids	NRP1	Thalamic reticular nucleus neurons	[74, 98, 99]
	ECEL1	Thalamic reticular nucleus	
	TCF7L2	Thalamus‐specific marker protein	
Hypothalamic organoids	RAX	Hypothalamic preoptic area neurons	[100, 101]
	OTP	Hypothalamic neural progenitor cells	
	NKX2‐1	Ventral hypothalamic neurons	
	POMC	Hypothalamic paraventricular nucleus neurons	
Striatal organoids	DARPP32	Striatal medium spiny neurons	[102, 103, 104]
	CTIP2	Striatal neurons	
Choroid plexus organoids	TTR	Choroid plexus epithelial cells	[50, 53, 54, 105]
	CLIC6		
	MSX1		
Pituitary organoids	ACTH	Adrenocorticotropic hormone‐secreting cells	[100, 106, 107]
	TBX19	Corticotroph progenitors, mature corticotrophs	
	PRL	Prolactin‐secreting cells	
	PITX1	Pituitary progenitor cells and anterior pituitary hormone‐secreting cells	
Retinal organoid	CHX10、VSX2	Retinal bipolar cell	[108, 109] [4, 110, 111]
	RHO	Rod photoreceptors	
	BRN3A	Retinal ganglion cells	
	MITF	Retinal pigment epithelial cells	
	CRX	Photoreceptor precursors, mature photoreceptors	
	GS	Müller glia cells	
Spinal cord organoid	PAX6	Ventral spinal neural progenitors	[28, 112, 113]
	FOXP1	Spinal cord interneurons	
	ISL1	Spinal cord motor neurons	
	OLIG2	Oligodendrocyte precursor cells and mature oligodendrocytes	

### 2.3 Strategies for Enhancing the Maturity of Neural Organoids

**FIGURE 4:**
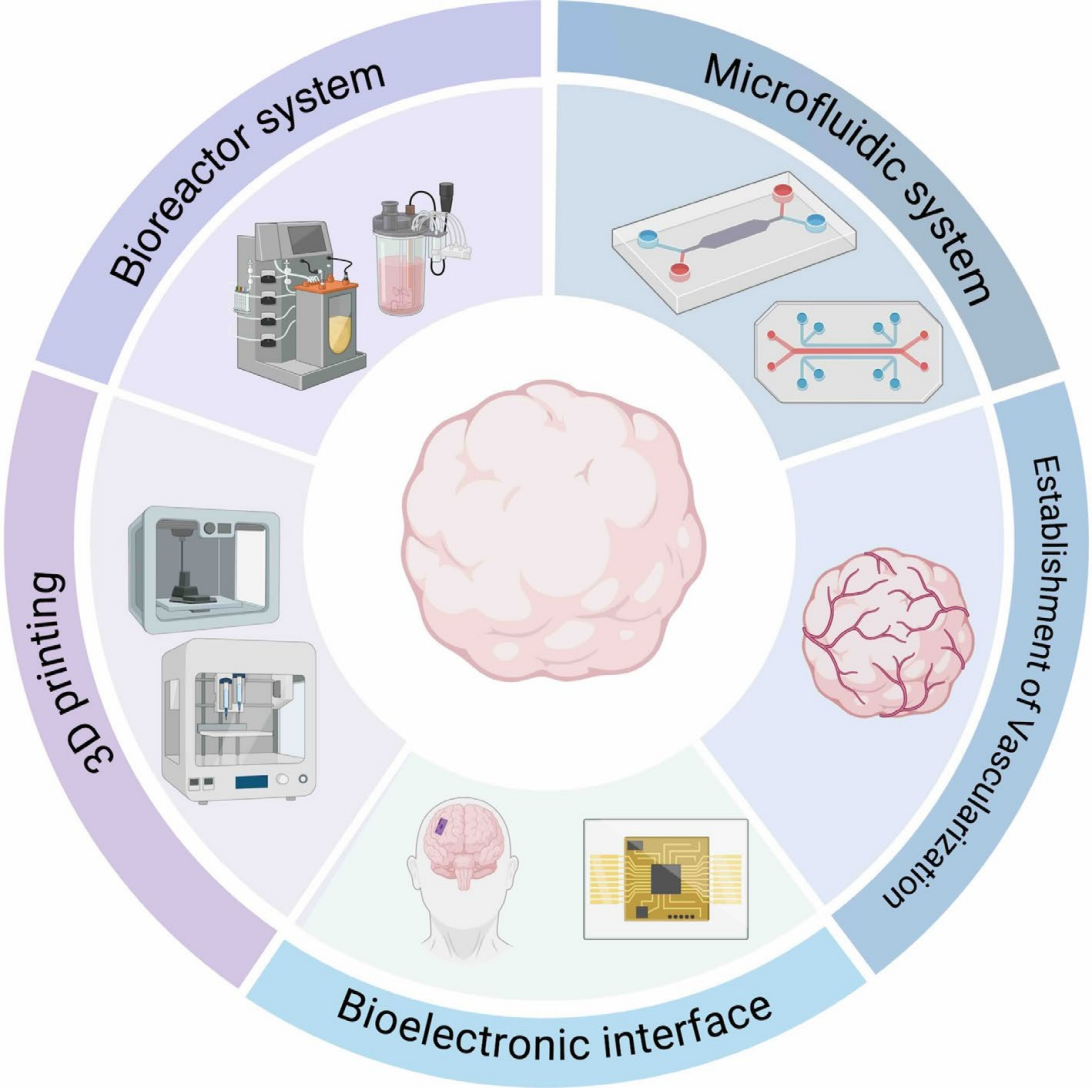
Maturity enhancement strategies.

Limitations and drawbacks encountered during organoid culture include the following: (1) necrosis in the core region due to hypoxia; (2) slow transport of nutrients and delayed clearance of metabolic wastes; (3) lack of mechanical stimuli and shear stress, which impairs neural regeneration. Under these drawbacks, organoids ultimately exhibit restricted growth potential. With the continuous advancement of biotechnology, various strategies and technologies have been explored to optimise the generation and functionalisation of organoids, including bioreactor systems, microfluidic systems, 3D bioprinting, bioelectronic interface, and the development of vascularised organoids (Figure 4). Innovations in these technologies are addressing the challenges in organoid culture, holding promise for generating complex organoids and providing a biologically simulated microenvironment for human neuron development and regenerative medicine.

#### 2.3.1 Bioreactor Systems

Bioreactors could promote the absorption of oxygen and nutrients by regulating the bioreactor operating parameters and physical forces of the extracellular matrix, thereby providing low fluid shear stress to facilitate intercellular interaction, differentiation induction, and the regulation of intracellular signal transduction and gene expression [114]. Additionally, bioreactor systems enable standardised mass production of neural organoids, addressing the low reproducibility issue of organoids in clinical applications. This helps establish standards for neural organoids in clinical use and facilitates quantitative modeling of brain diseases and compound testing.

Lancaster and Knoblich developed a rotating bioreactor, leveraging advances in pluripotent stem cell technology [115]. The key component of this bioreactor is agitation, which improves the diffusion of oxygen and nutrients and facilitates the formation of neuron organoids [116]. Another study demonstrated that using rotating‐wall vessel bioreactors can accelerate and improve organoid growth and differentiation [117]. When retinal organoids were cultured using rotating‐wall vessel bioreactors, they exhibited a very high level of differentiation, including the formation of ganglion cells and S‐cone photoreceptors [118]. Another group adopted the perfusion bioreactor that enables stable nutrient flow and differentiation to control the culture microenvironment of neuronal cells, while removing toxic by‐products. In long‐term cultures, laminar flow with low hydrodynamic shear stress offers advantages for the development of neural networks in vitro [5].

In contrast to orbital mixing, the organoids generated by vertical‐mixing bioreactors showed neurons that migrated from the outer periphery to the inner core [6]. Vertical mixing maintains high turbulent energy around the organoids and continuously disperses and adds uniform rheological force to keep inter‐organoid distances [6]. This indicated that mechanical forces applied through biomechanical engineering can influence the structural formation of brain organoids. The weakness of rotating bioreactor systems was their requirement for large volumes of culture medium and substantial cultivation space [119]. To address this limitation, Spin Ω had been developed as a miniature porous rotating bioreactor [120]. Researchers utilised the Spin Ω system to generate specific organoids from hiPSCs, including forebrain, midbrain, and hypothalamic organoids [120]. Through miniaturisation and increased throughput, it enhanced the efficacy of growth factors, enabling reliable and quantifiable production.

Recently, the rotating cell culture system microgravity bioreactor has achieved approximately 95% harvestability with enriched cellular diversity and structural brain morphogenesis, demonstrating high levels of physiological fidelity [121]. Another study demonstrated that integrating a mesofluidic bioreactor device with longitudinal tracking and machine learning‐based classification tools provides researchers with an integrated platform for non‐invasive quality control of live organoids and the establishment of reproducible culture standards [122].

The standardisation of organoid culture production remains challenging due to various factors. Hongwei Cai's [123] team developed intelligent acoustofluidics‐based mini‐bioreactors that comprised three key components: rotors for contact‐free rotation, a real‐time tracking module, and a reinforcement learning‐based controller. When integrated with machine learning, these automated bioreactors delivered more standardised and optimal performance while enhancing the efficiency and uniformity of organoid neural differentiation [123].

With the rapid advancement of biotechnology, increasingly miniaturised, cost‐effective, and high‐throughput bioreactor systems are being developed for the robust generation of brain organoids. These technological innovations will facilitate the clinical standardised production of neural organoids, thereby accelerating the translational pipeline from fundamental research to clinical application.

#### 2.3.2 Microfluidic Systems

In microfluidic devices, precisely designed microchannels, microvalves, micro mixers and other structures enable precise regulation of fluid velocity, pressure, and component concentration in the culture system [124]. This precise control allows highly accurate simulation of the dynamic characteristics of cerebrospinal fluid circulation, nutrient gradient distribution, and cellular signalling during in vivo brain development in organoid culture, thereby fundamentally improving the maturity, homogeneity, and functional integrity of brain organoids [125].

As in the developmental process in vivo, the differentiation of motor neurons is largely driven by chemical gradients and occurs in a concentration‐dependent fashion. Microfluidic devices can apply concentration gradients to 3D hydrogel‐based cell culture systems. Using computational and fluorescent dextran‐based characterisations, we could clearly observe the formation and stability of the orthogonal gradients [126]. During the development of the neural tube, these four signalling molecules (SHH, BMP, RA, and FGF) jointly coordinate the spatiotemporal differentiation and expression of cells. Microfluidic platforms, which operate based on the principle of Fickian diffusion, have been applied to mimic the precise spatiotemporal chemical environment required for neural tube development [127]. Through such platforms, the recapitulation of the spatial organisation of neural tube development has been observed in vitro.

To achieve a more physiologically relevant cerebral organoid culture, dynamic fluid perturbation was introduced into the 3D microfluidic platform to improve the culture quality [6]. Temporal proteomic analysis identified 546 proteins whose expression changes differently accompanying cerebral organoid development, relating to nervous system development and migration [128]. Microfluidic technology allowed a system to be constructed in which cortical, hippocampal, and thalamic organoids not only grow and function well, but also exhibit characteristics such as active neural migration and interaction. As reported, the microfluidic NT‐like structure derived from hPSCs had the potential to recapitulate neural patterning along the rostral‐caudal and dorsal‐ventral axes. It could also offer in vivo‐like spatiotemporal cell differentiation and organisation within 3D architectures [129]. Besides, the multichannel microfluidic chip was used to investigate the neurotoxic effects of low‐dose, constant BPS exposure on cerebral organoids, providing a generalised platform for studying the impact of various hazardous chemicals on these organoids [130].

Combining microfluidic technology with brain organoid technology, brain organoid chips are constructed to control the flow and mixing of fluids at the micrometre scale [131]. Microfluidic organs‐on‐a‐chip technology created a microenvironment similar to that of the human brain and realises the simulation of brain physiological functions and disease states [132]. Research indicated that microfluidic chips enhanced nutrient delivery and reduced necrosis in the organism's core, thereby increasing neural differentiation [133]. Another study combined iPSC and micro‐engineered organ‐chip technologies to establish a novel personalised platform of the human blood–brain barrier that could successfully model disease and screen drugs [134]. Microfluidic chip technology enables the real‐time study of human cells in an engineered physiological microenvironment [7].

Through microscale fluid manipulation, gradient construction, intercellular interaction regulation, and standardised design, microfluidic technology not only simulates the dynamic microenvironment of in vivo brain development in principle, but also addresses the core drawbacks of traditional culture in mechanism [135]. Meanwhile, its multi‐directional expanded applications provide reliable technical support for the efficient and precise production, basic research, and clinical translation of brain organoids [8].

#### 2.3.3 Vascularised Neural Organoids

During long‐term culture, brain organoids grew to a size of nearly 4 mm, which makes it difficult for oxygen and nutrients to diffuse into their interior. This leads to hypoxia and necrosis of cells inside the organoids, preventing them from further growth and development [50]. Although brain slicing technology could deliver oxygen and nutrients to deep brain organoid layers, it inevitably damages their inherent 3D architecture—a key feature mimicking in vivo brain spatial complexity [136]. Establishing vascularisation in vitro is of great importance for both the development of more physiologically relevant human neuron models and research in regenerative medicine [137].

One of the methods to generate vascularised brain organoids is to transplant brain organoids into the brains of rodents. The grafts could form glial integration and functional synaptic connections with the host [138]. However, variations in gene expression profiles across species may affect the development of brain organoids. Endothelial and parietal cell populations promote vascularisation in brain organoids. Vascularised organoids generated by the in vitro co‐culture of human umbilical vein endothelial cells with hESCs or hiPSCs possessed a functional vascular system. After transplantation, these organoids could integrate into the host's cortical tissue [79]. Furthermore, transplantation of vascular cells into cortical organoids not only facilitated the formation of vascular networks but also enhanced neurogenesis within cortical organoids while reducing cellular stress levels [139]. Another study found that the microfluidic cerebral organoid‐vascular bed co‐culture system could produce cerebral organoids on pre‐formed blood vessels [140]. Using CYR61 and HDGF as modifiers could improve the vascularisation and angiogenesis of neuron organoids [141].

Recent studies provided reliable approaches for generating vascularised human brain organoids. Vascular organoids can recapitulate the structure and function of developing human blood vessels [142]. The incorporation of vascular organoids into brain organoids is a reliable method for generating vascularised human brain organoids. Through co‐culture, a structure similar to native blood vessels was formed, where endothelial tubes were surrounded by mural cells and enclosed within a basement membrane [143]. Recently, using a custom‐designed 3D‐printed microfluidic chip to enable interactions between organoids and blood vessels was a simple and highly cost‐effective method for vascularising organoids [144].

The vascular system in organoids can efficiently transport oxygen and nutrients, clear metabolic wastes, and provide crucial cues for neural development to guide processes such as neural cell differentiation and migration. Such vascularised brain organoids are more analogous to the physiology of the human brain and exhibit crucial value and broad application potential in the field of regenerative medicine, brain injury repair [145].

#### 2.3.4 3D Bioprinting

3D bioprinting technology utilises bioinks composed of living cells and supportive hydrogel matrix to precisely fabricate functional three‐dimensional organoids under specific conditions, so as to simulate the microenvironment of natural tissues and organs [12]. The core of 3D bioprinting technology lies in its ability to precisely control the spatial position and microenvironment of cells, providing an unprecedented level of controllability for the research of neural organoids and establishing a standardised and highly controllable platform for such research [146].

In the 3D printing of neural organoid, polysaccharide‐based bioinks, which are composed of alginate, carboxymethyl‐chitosan, and agarose, are used, which can realise the construction of three‐dimensional scaffolds and rapid gelation [147]. Diluting the hydrogel in a solution of sodium alginate and carboxymethyl cellulose enabled it to print the generation of cortical organoids derived from iPSCs [148]. When the cell source is replaced with neural precursor cells and astrocytes, a functional neuron‐astrocyte network can be constructed by printing. Astrocyte precursor cells further developed into mature astrocytes, leading to the formation of a functional neuron‐astrocyte network [12]. Automated 3D bioprinters were capable of rapidly producing multiple organoids with high reproducibility [149]. In another study, high‐throughput, tunable, and reproducible scaffolds were generated, which can regulate and control the formation of organoids. During the co‐culture of organoids and blood vessels, endothelial cells were found to migrate and integrate into brain organoids [11]. Generated from two‐photon polymerisation 3D printing technology, retinal blood vessels could achieve biocompatibility with brain organoids during long‐term co‐culture [150]. In the cultural process, there are limitations such as the transfer of cultural platforms and the excessive use of plastic products. A 3D‐printed culture workflow had been developed, which enables all steps to be carried out in a single well plate. Moreover, this platform has been used to achieve the culture of human ventral midbrain organoids [151].

Using iPSC‐derived spinal neuronal progenitor cells and oligodendrocyte progenitor cells as raw materials, a bioengineered spinal cord was constructed via extrusion‐based multi‐material 3D bioprinting technology, which can reestablish functional axonal connections in the injured area of the CNS [152]. 3D‐printed scaffolds fabricated via the microscale continuous projection printing method possessed the ability to support axonal regeneration and create a complex CNS structure for regenerative medicine applications in the spinal cord [153].

In conclusion, 3D bioprinting enables the precise spatial positioning of cells and matrices, which is expected to achieve the accurate and controllable production of neural organoids and promote the standardisation of their translation into clinical applications [20]. Meanwhile, with the continuous advancement of 3D bioprinting technology, there is also a need to establish a comprehensive and flexible ethical regulatory framework to guide its responsible development [154].

#### 2.3.5 Bioelectronic Interfaces

In the nervous system, functionally active neurons form neural networks and generate bioelectrical activities. Similarly, brain organoids can simulate the nervous system in vitro and develop electrophysiological activities. Leveraging the high fidelity, specificity, and biocompatibility of bioelectronic interfaces enabled the achievement of highly uniform and reliable synchronous recordings of brain electrical activities [155]. By detecting such activities, the physiological maturity and safety of organoids could be assessed, and the establishment of standardised quality for neural organoids could be promoted [156].

On one hand, bioelectronic interfaces can be used for the dynamic monitoring of neural organoids to collect electrophysiological data [157]. During long‐term culture of neural organoids, microelectrode array devices allow for non‐invasive detection of electrophysiological networks, which helped identify abnormalities in electrophysiological activity [158]. 3D liquid metal microelectrodes, with their mechanical flexibility, allowed comprehensive electrophysiological analysis of retinal organoids while minimising damage [110]. Additionally, stretchable electrode arrays could adapt to changes in the volume and shape of brain organoids, enabling long‐term, stable, and continuous recordings [159].

Furthermore, bioelectronic interfaces can be utilised to detect organoids after transplantation. Studies have demonstrated that xenografted neurons can integrate into the mouse cortex and establish strong electrophysiological connections [160]. Graphene microelectrode arrays, which provide flexibility and optical transparency, enabled long‐term monitoring of the integration between the graft and the host neural network when cortical organoids were co‐implanted with these arrays into the mouse cortex. However, long‐term implanted microelectrodes encounter interference from neuronal activities during information processing, and this remains a major challenge for future research [161].

Beyond electrophysiological detection, electrical stimulation has been confirmed to influence neural development and regulate neural networks [162]. Applying electrical stimulation via multi‐electrode arrays to deliver bioelectrical signals into cortical organoids could promote their differentiation and maturation, resulting in enhanced activity and maturity after transplantation [163].

This electrical stimulation technology is expected to advance the development of next‐generation neural organoids with higher physiological activity and maturity. It can also facilitate the establishment of standardised quality control systems for neural organoids. Thus, it represents a promising direction in neural organoid transplantation research [164].

## 3 Neural Organoid Transplantation for CNS Repair and Regeneration

### 3.1 Neural Organoid Transplantation

The CNS exhibits limited regenerative capacity. When subjected to irreversible damage, it cannot rely on its intrinsic growth ability to achieve self‐repair. Currently, organ transplantation remains the primary therapeutic approach for irreversible CNS injuries. However, due to the scarcity of donor organs and the risk of immune rejection, a large proportion of patients still fail to receive effective treatment. Against this backdrop, the transplantation of brain organoids offers a promising prospect for treating otherwise incurable CNS diseases, providing a repair strategy that is more physiologically relevant.

**FIGURE 5:**
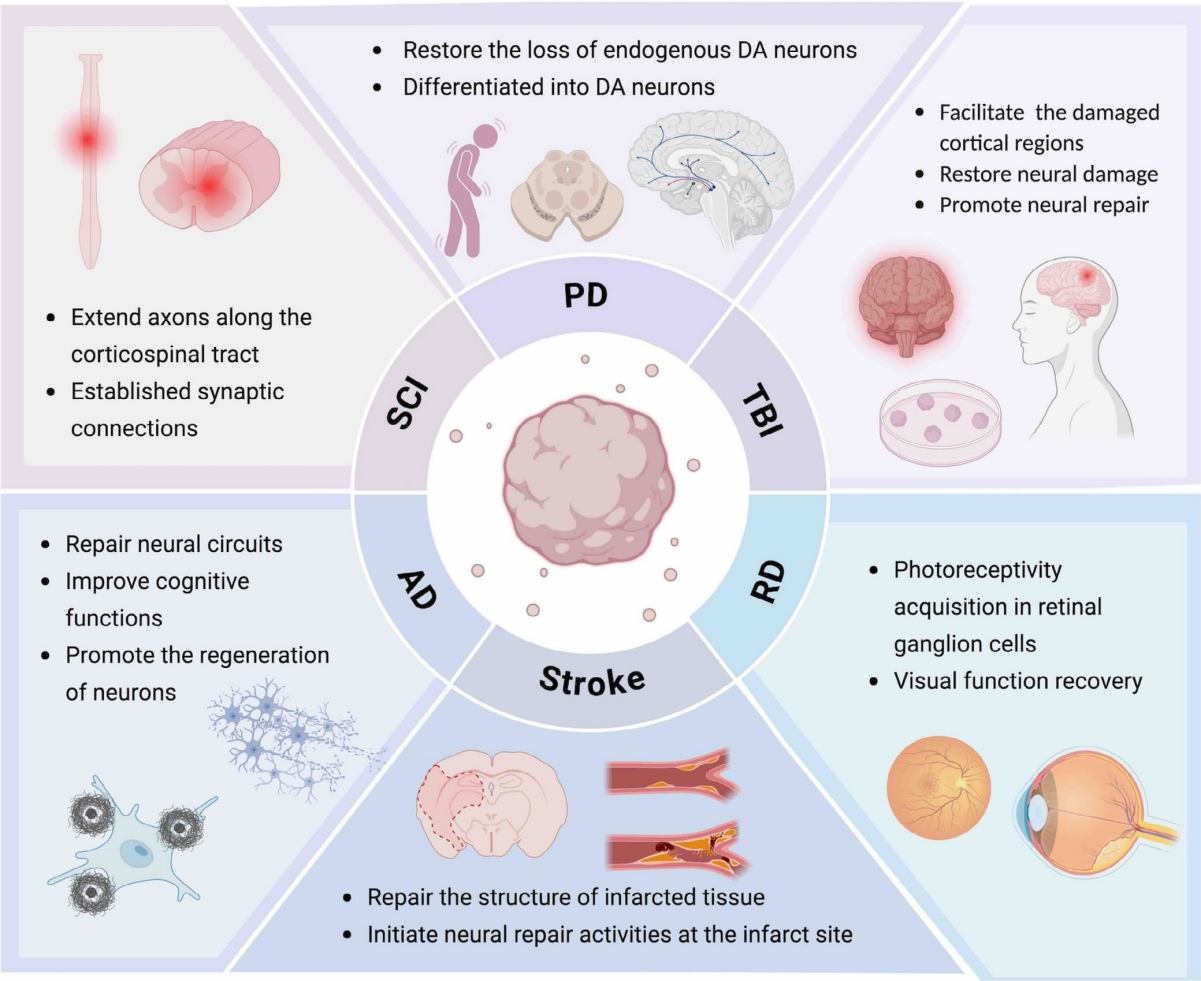
Application of neural organoids and their extracellular vesicles/exosomes in transplantation. AD, Alzheimer's disease; PD, Parkinson's disease; RD, retinitis pigmentosa; SCI, spinal cord injury; TBI, traumatic brain injury.

To date, a variety of neural organoids have been generated, such as cerebral organoids [165], midbrain organoids [166], forebrain organoids [52], cortical organoids [79], pituitary organoids [167, 168], spinal cord organoids [76, 169], retinal organoids [170]. These organoids have shown promising potential in transplantation and regenerative therapy for CNS diseases, including TBI, stroke, Parkinson's disease (PD), SCI, retinal degenerative diseases, and amyotrophic lateral sclerosis (Figure 5). Subsequently, we will focus on analyzing the repair and regenerative effects of neural organoids on TBI, stroke, PD, SCI, and retinal degenerative diseases (Table 2).

**TABLE 2:** The different neural organoids from iPSCs/ESCs, MEFs and CiPSCs or human astrocytes used for transplantation and regeneration.

Application	Type of organoid	Transplantation information	Achievements
TBI	Cerebral organoids	hESC, SD rats, motor cortex	Cortical differentiation of transplanted cerebral organoids revealed by TBR1/SATB2 immunostaining [171]
		hESC, SCID mice, left parietal cortex	Extension of long projections and spontaneous activity, induced vascularisation [172]
		hESC, C57BL/6 J mice, cortical cavity	Repair of the damaged cortical region and hippocampal injury [165]
		hiPSC, SCID mice, frontal motor cortex	Enhancement of transplantation efficiency and notable increase in cortical projection neurons [173]
		hESC and hiPSC, SCID mice, prefrontal cortex and hippocampus	Higher maturity of astrocytes transplanted in the PFC [174]
Stroke	Cerebral organoids	hESC, rats, motor cortex	Reduction of cerebral infarct volume, promotion of neurogenesis, and angiogenesis by transplantation [175]
		hESC and hiPSC, NOD‐SCID mice, ischemic core	Repair of infarcted tissue, restoration of infarcted tissue structure and elimination of sensorimotor deficit behaviours [176]
		hESC, SCID mice, bilateral frontal and parietal cortices, purpose‐bred male cynomolgus monkeys, primary motor cortex	Axon extension along the CST, potential for reconstruction of the host motor pathway [177]
			Provision of axonal extension along the corpus callosum and subcortical projections [177]
Parkinson's disease	Human midbrain organoids	hiPSC and hESC, rats, striatum	Synaptic maturation of Og‐NSC‐derived mDA neurons after transplantation under the support of astrocytes [178]
		hiPSC, SCID mice, striatum	Survival and maturation of transplanted cells, and functional recovery of motor function [179]
		iPSCs, mice, medial forebrain bundle	Endogenous dopamine release measured by GRABDA2h in striatal slices for neural network integration [166]
		hiPSC, SD rats, substantia nigra	Differentiation into dopaminergic lineage neurons and improvement of motor dysfunction [180]
SCI	Cerebral organoids	hiPSC, SD rats, spinal cord (T10 levels)	Acquisition of more regenerated nerve fibres and presence of activated microglia [181]
	Spinal cord organoids	human astrocytes, NOD‐SCID mice, spinal cord (T9–T10 levels)	Co‐localisation of human synaptophysin^+^ synapses and ChAT^+^ host motor neurons revealing synapse formation [76]
		hiPSC, SD rats, spinal cord (T10 levels)	Promotion of neuronal survival and integration, and alleviation of glial scar formation [169]
		Neural progenitors, nude mice, spinal cord (T10 levels)	Enhancement of motor function recovery, reduction of glial scar formation, and guidance of axonal regrowth through its channel‐patterned architecture [182]
		iPSC, NOD mice, spinal cord (T9 levels)	Mimicry of the thoracic spinal cord tissue pattern and enhanced thoracic segment‐specific tissue [183]
Retinal degenerative diseases	Retinal organoids	hESC, RCS rats, subretinal space of the left eye	Long‐term survival and improvement of visual function, generation of new photoreceptors [170, 184]
		MEFs and CiPSCs, C57BL/6J mice, subretinal space	Integration into host retina and synaptic connection establishment revealed by co‐localisation of synaptophysin and CtBP2 [185]
		hiPSC, hESC, nude mice, subretinal space	Long‐term survival and trophic support of human iPSC‐derived retinal cells in the host [186, 187, 188, 189]
		hiPSC, C57BL/6 mice, vitreous cavity	Detection of presynaptic markers and postsynaptic markers to confirm synaptic structural integration [190, 191]
		iPSC, human, subretinal space	Safety and stable survival rate demonstrated within 2 years [192]
		hESCs, nude rats, subretinal space of the left eye	Promotion of the survival, maturation and reconstruction, enhancement of visual function improvement [193]
Generation of vascularised organoids	Cerebral organoids	hESC, CD1 mice, frontoparietal cortex	Higher post‐transplantation survival rate and robust vascularisation [194]
	Cerebral organoids	hESC, NOD‐SCID mice, retrosplenial cortex	Functional synaptic integration between brain organoids and host revealed by immunostaining and electrophysiological recording [195]
	Cortical organoids	hESC and hiPSC, NOD‐SCID mice, S1 cortex	Construction of functional human‐mouse chimeric blood vessels [79]
Hypopituitarism	Pituitary organoids	hiPSCs, SCID mice, pituitary	Retain both positive and negative feedback mechanisms, recapitulating mature endocrine organs in vivo [167]
		hESC, SCID mice, inguinal subcutaneous white adipose tissue and beneath dorsal skin	Expression of angiogenic factors, autonomous promotion of vascularisation and transplantation [106]
	Pituitary–hypothalamic organoids	hiPSC, SCID mice, renal capsule	High adrenocorticotropic hormone (ACTH) secretory capacity and responded to both positive and negative regulators [168]
Cortical damage	Forebrain organoids	hiPSC and hESC, Long Evans rats, cortical cavity	Restoration of visual function following lesions [51, 196]
Functional integration of neural circuits	Cerebral organoids	hESC and hiPSC, SCID mice, medial prefrontal cortex	Subcortical projections formed between human cerebral organoids and the host [197]
	Cortical organoids	hiPSC, NOD/SCID mice, retrosplenial cortex	Functional integration between the organoid and the host brain using multimodal monitoring techniques [198]
The fidelity of organoid models	Cortical organoids	hiPSC, NSG mice, cortex	Ramework for evaluating and improving the accuracy of cortical organoids [95]
The host circuits involved in behavioural control	Cortical organoids	Human stem cell, newborn athymic rats, somatosensory cortex	Maturation, axonal projection extension and integration of transplanted human cortical neurons via immunostaining, electrophysiological recording and behavioural testing [156]
Glial cell‐enriched cortical organoid model	Cortical organoids	hESC and hiPSC, NSG mice, hippocampus	Maturation and differentiation of astrocytes in transplanted glia‐enriched cortical organoids [199]
Establishment of transplantation methods	Cortical organoids	hiPSC, nude rat, somatosensory cortex	Effective combination of cortical organoids with host neural circuits and their participation in host neural signal transmission using optogenetics and behavioural testing techniques [200]

Abbreviations: hESC, human embryonic stem cells; hiPSC, human induced pluripotent stem cells; iPSC, induced pluripotent stem cells; SCI, spinal cord injury; TBI, traumatic brain injury.

#### 3.1.1 Traumatic Brain Injury

TBI refers to damage to the brain caused by intense external physical forces, resulting in brain tissue deformation and changes in intracranial pressure. Each year, TBI impairs the healthy lives of millions of people worldwide. Based on the Glasgow Coma Scale, TBI can be classified into mild, moderate, and severe grades [201], imposing distinct physiological, psychological, and economic burdens on patients [202]. Additionally, psychiatric and cognitive impairments that accompany TBI after injury compromise patients' cognitive function and quality of life [203]. Pathologically, excitotoxicity induced by TBI leads to excessive neuronal stimulation and synaptic loss, ultimately resulting in cell death [204]. Currently, there are no drugs capable of reducing or repairing brain damage caused by TBI. Brain organoids offer a promising regenerative medicine strategy. By transplanting them to replace functional neural cells in injured regions, they have the potential to repair neural injuries [205].

In previous studies, cell transplantation approaches have been limited by poor post‐transplantation cell survival and insufficient neuronal differentiation. In contrast to the transplantation of neural progenitor cells, the transplantation of brain organoids derived from hPSCsdemonstrated higher survival rates in the host brain, highlighting their potential for neural repair [194]. Concurrently, following transplantation, the host exhibited robust neural differentiation, synaptic connections, and extensive vascularisation, ultimately achieving functional integration between the graft and the host [195]. In mild TBI, cerebral organoid transplantation facilitates the reconstruction of damaged cortical regions, restores neural damage in the dentate gyrus regions of the hippocampus, and alleviates cognitive impairments [165]. Similarly, in mice with TBI and severe combined immunodeficiency, implanted brain organoids undergo multi‐lineage differentiation. These reduced glial scarring, improves the local microenvironment, and promotes neural repair. Consequently, deficits in spatial learning and memory in these mice were ameliorated following organoid transplantation [172].

Beyond these findings, a study by Revah et al. [156] revealed that transplanted cortical neurons from organoids receive and integrate axonal inputs from sensory‐related circuits and cortical projections. These neurons extend axonal projections throughout the rat brain, and their optogenetic activation is capable of driving reward‐seeking behaviors.

Brain organoids at different developmental stages vary in neural cell types, regional characteristics, and cell quantity. To identify which developmental stage of brain organoids was suitable for transplantation, the research team selected 55‐day‐old cerebral organoids and 85‐day‐old cerebral organoids for transplantation experiments, respectively. It has been shown that 55 d‐COs are superior to 85 d‐COs as transplant donors: after transplantation into the damaged motor cortex, they exhibit higher rates of neuronal survival and generation, and support the region‐specific reconstruction of the injured motor cortex [171]. However, transplantation of early‐developing brain organoids for brain injury treatment also carries the risk of excessive growth of neurons and axons, which may pose potential safety concerns [177].

Modulating the host brain microenvironment represents a viable strategy to promote axonal extension of transplanted brain organoids. A 1‐week delay between lesion induction and transplantation significantly enhances vascularisation, survival, and proliferation of transplanted cells. This delay also substantially increases the density of projections formed by transplanted neurons, thereby improving functional repair [206]. Gene expression analysis revealed that, compared with immediate transplantation post‐injury, a 1‐week delay creates a more favorable host microenvironment [173]. Specifically, delaying transplantation by 1 week after cerebral cortex resection enhances both the survival rate of transplanted 10‐week‐old brain organoids and their axonal extension [177]. Additionally, studies have shown that supplementation with the exogenous neurotrophic factor recombinant human progranulin [207, 208] can mimic the effects of this optimal host microenvironment, thereby enhancing the therapeutic efficacy of transplantation [173].

#### 3.1.2 Stroke

Stroke is one of the leading causes of disability and impairment worldwide, ranking as the second most common cause of death among non‐communicable diseases [209]. Data from *The Lancet* indicate that the number of stroke‐related deaths is projected to increase by 50% from 2020 to 2050 globally [210]. Stroke directly results in neuronal loss and excessive production of reactive oxygen species, which exacerbate neuronal damage and impair the stability and integrity of brain function. Over the past few decades, numerous neuroprotective drugs have been investigated, yet they remain in the transition from preclinical research to clinical application. Currently, the only FDA‐approved therapeutic agent for stroke is tissue plasminogen activator [211], but its use yields minimal benefits for neurological recovery. Traditional treatment approaches, such as intravenous thrombolysis [212] and endovascular thrombectomy [213], can alleviate patients' conditions. However, these methods fail to truly repair damaged neural tissue or restore neurological function, and they still carry the risk of intracerebral haemorrhage. Thus, there is an urgent need to develop stroke treatment strategies focused on neural repair and regeneration.

Neurological repair after stroke is a complex process involving synergistic multiple mechanisms and multifactorial regulation. In addition to neurogenesis, neural plasticity, angiogenesis, and tissue reconstruction mediated by organoid transplantation [156], hemispheric compensatory mechanisms serve as an important endogenous regulatory pathway for functional recovery post‐stroke [214]. External environmental factors such as exercise and social interaction can modulate the efficiency of hemispheric compensation by reshaping brain structure and functional networks [215, 216]. Meanwhile, these external factors also directly influence cell survival, differentiation, integration, and functional reconstruction after organoid transplantation, acting as a critical bridge linking endogenous repair and exogenous intervention [217].

Transplantation of neural stem cells to replace lost neuronal function shows promise for functional recovery after stroke [218]. Transplanted neurons can differentiate and integrate into the brain's neural circuits, contributing to injured tissue repair through neurogenesis, neurotrophic factor production, new cell generation, and regulation of immune responses [219]. However, cell therapy has inherent limitations: transplanted neurons are overly dispersed, failing to truly repair lost tissue and accompanied by safety risks [220].

In contrast to cell transplantation, neural organoids possess a substantial cell quantity, diverse neural cell types, tissue‐like morphological features, enhanced survival and integration capabilities, and form a complete microenvironment. After a stroke, infarcted tissue was formed, and neuron organoid transplantation can repair the structure of infarcted tissue and initiate neural repair activities at the infarct site [221]. Reconstructing brain connectivity is critical for restoring the integrity of neural networks and brain function. Using brain organoids and an agarose shell, constrained 3D human axon tracts could be generated. These tracts consist of distinct cellular regions and allow for physical manipulation [222]. Currently, the middle cerebral artery occlusion model in mice is the most widely used for organoid transplantation studies [223]. Cerebral organoids were transplanted 6–24 h after stroke modelling and differentiated into multiple cell types, supporting the specific reconstruction of the motor cortex and establishing synaptic connections with the host brain. Cerebral organoids transplantation reduces cerebral infarct volume, decreases neural cell apoptosis, and improves post‐stroke neuromotor function.

When brain organoids derived from hPSCs were transplanted into the boundary between the infarct core and peri‐infarct region of stroke‐induced NOD‐SCID mice, the grafts exhibited distinct differentiation states in these two regions. In the infarct core, over 65% of organoid‐derived cells were glial cells, which can fill infarct tissue defects and improve the microenvironment. In the peri‐infarct region, glutamatergic neurons predominated, with only a small number of astrocytes and oligodendrocytes. GABAergic neurons are the dominant inhibitory neurons that regulate neural circuits and neural activity. Loss of GABAergic neurons after stroke may lead to sensorimotor deficits. When human midbrain GABAergic organoids derived from hiPSCs were transplanted into the infarcted cortex of stroke mice, the transplanted organoids predominantly differentiated into GABAergic neurons and significantly restored sensorimotor deficits in stroke mice [224].

Combined with bioengineering, magnetic resonance imaging could be used to scan the damaged area, and 3D scaffolds with specific sizes and shapes can be fabricated to provide a favourable environment for cell migration during stroke repair [225]. These studies provided first‐hand preclinical evidence for cerebral organoids as a potentially effective intervention for stroke treatment, holding promise for achieving precise reconstruction of damaged neural tissue and functional recovery, thereby addressing the limitations of traditional therapies.

#### 3.1.3 Parkinson's Disease

Parkinson's disease (PD) is a common neurodegenerative disorder and one of the major health threats in aging societies, affecting over 10 million people worldwide. In terms of pathological features, patients with PD exhibit significant degeneration and death of dopaminergic (DA) neurons in the substantia nigra pars compacta of the brain, which leads to a marked reduction in dopamine release in the striatum [226]. As DA levels decline, patients develop the characteristic motor symptoms of PD, including rigidity, bradykinesia, and postural instability, along with non‐motor manifestations.

Clinically, standard treatments for PD include levodopa administration and surgical deep‐brain stimulation. However, these therapeutic approaches only provide temporary relief of symptoms and fail to truly restore or compensate for the degenerated neurons. In contrast, cell therapy has emerged as a promising strategy for the treatment of PD [227]. Exploring hiPSCs or hESCs, these stem cell‐derived DA neurons were capable of replenishing and restoring the loss of endogenous DA neurons and have been shown to significantly ameliorate motor deficits in PD mouse models [228, 229]. Clinical trials have also demonstrated improvements in safety profiles, non‐pharmacological motor function, and transplantation survival rates [230].

Additionally, by first generating midbrain organoids (hMOs) from hESCs or hiPSCs, and then isolating neural stem cells from the midbrain organoids, these cells could be stably expanded and efficiently differentiated into DA neurons. These DA neurons release dopamine and integrate into the neural networks of PD mice, thereby improving the mice's motor function [178, 231]. Compared to single‐cell cultures, hMOs comprised multiple cell types and provided structural support, promoting graft survival, maturation, and rapid functional integration. When hiPSC‐derived hMOs were transplanted into the striatum of PD model mice, the survival and maturation of dopaminergic neurons were observed, and the motor function of the mice was successfully ameliorated [179].

In the experiment, it was observed that in the group receiving 3D cultured grafts, the recovery of motor function in mice began to be detected 6 weeks after transplantation. Meanwhile, no tumour formation or excessive graft overgrowth was detected in either group, indicating that hMOs hold promising application prospects [179]. Similarly, when hMOs were transplanted into the substantia nigra, the grafts survived long‐term, with over 80% of the grafted cells differentiating into tyrosine hydroxylase‐positive dopaminergic neurons. These TH^+^ neurons were capable of releasing dopamine, integrating into the host neural circuitry, and gradually reversing the motor deficits induced by 6‐hydroxydopamine lesions starting from the 5th week post‐transplantation. Ten weeks after implantation, no tumour formation or migration was detected in the subcutaneous space or major organs [180].

Using a stirred tank bioreactor, dopamine‐containing neurons derived from iPSCs were cultivated into neural microtissues. Following transplantation, mice exhibited complete functional recovery 16 weeks post‐transplantation [232]. In transplantation experiments, studies using non‐human primate PD models will provide more comprehensive information for organoid transplantation. In one study, brain organoids were transplanted into the brains of cynomolgus monkey models [177]. These organoids integrated into the host neural network, projected to the cortex, corpus callosum, and striatum, and promoted axonal elongation in the brains of cynomolgus monkeys [177]. While these studies were conducted using midbrain organoids, they provide confidence in the potential of implanting neuron organoids as a therapeutic approach for PD.

#### 3.1.4 Traumatic Spinal Cord Injury

Traumatic spinal cord injury (TSCI) leads to the loss of spinal cord tissue, including extensive neural cell and neural fibre loss, and thus results in the loss of motor and sensory functions, which seriously impairs patients' quality of life. Following TSCI, adult mammals exhibit limited neural regeneration capacity, with irreversible neural tissue loss that usually results in permanent lower limb dysfunction [233]. The complex pathological mechanisms and the difficulty of neural regeneration pose significant challenges to the repair and treatment of SCI. Clinically, therapeutic approaches for SCI include pharmacotherapy, surgery, and rehabilitation therapy [234]. With the development of regenerative medicine, increasing attention has been paid to the use of technologies involving stem cells and organoids to promote neural regeneration and neural circuit reconstruction [28, 235]. Previous research has indicated that mice with damaged spinal cord tissue can regain functional motor abilities following the transplantation of multiple neural cell varieties, including astrocytes, oligodendrocyte precursor cells, NSCs, and NPCs [236].

However, stem cells implanted into target tissues or organs exhibited a limited retention rate and transplantation efficiency after transplantation, resulting in stem cell transplantation outcomes that fail to meet expectations. The physiology and mechanical properties of the cerebral cortex and spinal cord tissue are similar. When 6‐week‐old cerebral organoids derived from hESCs were transplanted into the cerebral cortices of mice, they extended a greater number of axons along the corticospinal tract [177]. Following cerebral organoids transplantation, the regenerated nerve fibres of recipient animals increased, which in turn promoted tissue regeneration and neuronal connectivity [181].

In addition, human astrocytes can also be reprogrammed to form spinal cord organoids that contain functional neurons. Grafts of human‐astrocyte‐derived spinal cord organoids exhibited survival in recipient mice, differentiated into spinal cord neurons, extended long‐distance axons, established synaptic connections with host neurons, and exerted protective effects in a mouse model of complete SCI [76]. The integration of organoids with biomaterial scaffolds presents an innovative and promising regenerative approach [28, 182]. Integrating iPSC‐derived spinal cord organoids with GelMA hydrogel scaffolds significantly improves the host's structural and functional recovery, reduces glial scar formation, and promotes host‐graft synaptic integration, ultimately restoring functional neural circuits [169]. Recently, researchers engineered a thoracic vertebral segment‐specific spinal cord organoid. The transplantation of these organoids had enabled the reorganisation of neural circuits in paralysed mice and the restoration of hindlimb motor function, demonstrating robust neural functions and therapeutic efficacy, highlighting the effectiveness of organoid transplantation in the treatment of neural injuries [183].

As cutting‐edge technologies in the fields of stem cells and tissue engineering, the transplantation of cerebral organoids and spinal cord organoids provides a novel solution for advancing regenerative medicine therapies in the nervous system, with the potential to alleviate neurological deficits and restore function. Future research will focus on improving the maturity and functionality of cerebral and spinal cord organoids, as well as generating organoids that incorporate a more comprehensive developmental microenvironment.

#### 3.1.5 Retinal Degenerative Diseases

Retinal degenerative diseases (RDD) cause irreversible blindness and represent one of the primary causes of blindness worldwide. Pathologically, irreversible degeneration of retinal ganglion cells and retinal pigment epithelial cells leads to optic neuropathy, resulting in visual impairment. Moreover, the lack of repair or regeneration capacity after injury ultimately culminates in permanent loss of visual function.

3D retinal organoids self‐organised stem cells enable functional replacement, offering a promising therapeutic approach for retinal degenerative diseases. Early treatments for these diseases commonly relied on cell transplantation, such as retinal progenitors, immature photoreceptor progenitors [191], and mature progenitors [237], which can target and replenish specific lost cell populations. However, low survival rates and insufficient mechanical stability have limited the further application of this approach [238]. Retinal organoids possess an intact structure that provides mechanical support. They can improve the developmental microenvironment of the host retina and promote retinal cell differentiation and function [239].

When retinal organoids derived from iPSCs or hESCs were transplanted into model mice, the photoreceptors of the graft established connections with the host, inducing a photoresponse in the mice [191]. Supported by the tissue mechanical structure, the grafts exhibit higher post‐transplantation survival rates and achieve long‐term survival [187, 189]. Behavioural tests and electrophysiological assessments have demonstrated significant improvements in the visual function of the mice [184, 186]. Photoreceptor cells in retinal organoids are generally considered to play a crucial role [240]. Researchers have used enzymatic dissociation [241] or small molecule‐driven enrichment [242] to obtain high‐purity photoreceptor‐enriched organoids, which can integrate and differentiate in the host retina and restore function in mice with retinal degeneration. However, recent studies have revealed that horizontal cells can support the formation of host‐graft synapses and also contribute to retinal function recovery.

Additionally, the host microenvironment promotes the maturation of rod and cone photoreceptors derived from organoids as well as functional recovery after transplantation [243]. In clinical trials, transplantation of retinal organoids has also demonstrated safety and efficacy. One study showed that the graft survived in a stable state for 2 years, with increased retinal thickness at the transplantation site and no occurrence of serious adverse events [170]. Future research is needed to further verify the safety of retinal organoid transplantation in more cases, establish detailed visual function assessment methods, and confirm the connection between the graft and the host.

Furthermore, several combination therapy strategies have provided new insights for retinal organoid transplantation. Under the induction of small chemical molecules, 3D retinal organoids derived from canine iPSCs integrate into the host retina, establish synaptic connections, and significantly improve visual function in mouse models [185]. Hyaluronic acid enhanced photoreceptor commitment and differentiation in human retinal organoids, further promoting the generation and long‐term culture of retinal organoids [244]. Transplantation of gene‐edited retinal organoids improved the connection between host and graft photoreceptors and restored photosensitivity to a certain extent [241].

Using fibrin glue as a biological adhesive, retinal pigment epithelial cells and retinal organoids are co‐transplanted. This co‐transplantation strategy promoted the survival, maturation, and functional integration of transplanted photoreceptors, thereby enabling synaptic connections with host retinal neurons and achieving long‐term survival and functional integration after transplantation [170]. Three‐dimensional automated reporter quantification is used for screening complex stem cell‐derived retinal organoids. This method met the requirements of speed, sensitivity, and reproducibility for complex screening applications [57], offering prospects for future quality analysis of retinal organoids and reducing variability between organoids [245] and enabling standardised production of organoids [188].

#### 3.1.6 Requirements for Neural Organoid Transplantation in CNS Repair and Regeneration

Distinct pathological microenvironments in different neurological diseases lead to divergent requirements and priorities for neural organoid‐based therapies. The requirements for organoid integration that differ in different neurological diseases are summarised.

As for TBI, the injured microenvironment is characterised by glial scar formation and inflammation. The primary goals of neural organoid application are to suppress inflammation, fill tissue defects, and reconstruct neural circuits. Composite organoids containing neurons, astrocytes, and microglia are preferred to recapitulate the complex cellular composition of the brain and improve adaptability to the hostile microenvironment [246].

In PD, the core pathology involves progressive degeneration of dopaminergic neurons in the substantia nigra pars compacta [247]. The microenvironment exhibits dopamine deficiency and oxidative stress, with no obvious tissue cavitation [166]. Accordingly, neural organoids are required to secrete sufficient dopamine to compensate for the host's deficit, whereas vascular integration is not a major concern.

Stroke manifests as hypoxia, nutrient deprivation, and disrupted neural circuits, accompanied by abundant inhibitory signals surrounding the lesion cavity. Transplanted neural organoids must therefore be resistant to injury, hypoxia‐tolerant, and capable of vascular integration [248]. Ischemic brain organoid models are commonly established, and hypoxia‐resistant subpopulations are selected via in vitro hypoxic preconditioning [249]. Unlike PD, the stroke cavity lacks chronic progressive neurodegeneration but presents severe hypoxia and nutrient shortage; thus, organoid integration depends more strongly on vascularisation and hypoxia tolerance, whereas PD relies on long‐term functional stability.

SCI is characterised by severe axon disruption, glial scar obstruction, and persistent inflammation, which block neural signal transmission. For neural organoid transplantation in SCI, the key point is to overcome the barrier of axon outgrowth to promote axonal extension and synaptogenesis between grafted organoids and the host spinal cord [250].

RDD are driven by progressive degeneration of retinal photoreceptors and structural breakdown of the retina [251]. The microenvironment is marked by photoreceptor loss, impaired retinal barriers, and insufficient nutrient supply, with the primary functional goal of restoring visual photoreception [252]. Retinal organoids are typically generated, with directed differentiation towards photoreceptors and retinal pigment epithelial cells, and must maintain a well‐organised layered retinal structure [108]. In contrast to PD, where integration relies heavily on the functional maturation of dopaminergic neurons, RDD organoid integration depends more on structural lamination and photoreceptor specificity.

### 3.2 Neural Organoid–Derived EVs/Exosomes Transplantation for CNS Repair and Regeneration

To date, substantial findings have been accumulated in the research on organoid‐derived extracellular vesicles (EVs) and exosomes across various fields, including oncology, respiratory diseases, and orthopaedics, providing novel intervention strategies for refractory diseases [253]. While research on organoid‐derived EVs and exosomes in the neural field started relatively late, they have achieved a series of important discoveries by virtue of their unique advantages in neural tissue repair and regeneration. Particularly, they have demonstrated great application potential in the intervention of neuro‐related diseases, including PD, AD, and retinal diseases (Table 3).

**TABLE 3:** Neural organoid extracellular vesicles, exosomes, and their function.

Disease	Type	Therapeutic effect
Retinal degeneration	Retinal organoid–derived extracellular vesicles	EVs targeted regulation of the expression of approximately 60% of mammalian genes [254].
		EVs biogenesis biomarkers are highly expressed in the late stage of retinal organoids [255].
		EVs have superior antioxidant effect and show protective therapeutic effects on ARPE‐19 cells by targeting precise proteins linked to the activation of the AMPK pathway [256].
		EVs improve the lipid metabolism and protect retinal pigment epithelium cells against lipid cytotoxicity [108].
	Retinal organoids–derived exosomes	Exosomes increase in epithelial cell proliferation and migration of limbal stem cells, inhibition of inflammatory processes and regulation of substances related to wound healing [257].
		Exosomes reduced photoreceptor apoptosis, prevented outer nuclear layer thinning, and preserved visual function in RCS rats [258].
PD	Cerebral organoid–derived exosomes	Alleviation of oxidative stress, promotion of dopaminergic differentiation, and neuroprotective effect [259].
AD	Choroid plexus organoids–derived exosomes	Increase of biogenetic capacity with the developmental time of choroid plexus organoids [53].
ASD	Dorsal forebrain organoid–derived extracellular vesicles	Significant changes in RNA and protein 61 content in ASD EVs [260].

Abbreviations: AMD, age‐related macular degeneration; ASD, autism spectrum disorder; EVs, extracellular vesicles; PD, Parkinson's disease.

#### 3.2.1 Neural Organoid–Derived EVs/Exosomes

EVs are membrane‐bound nanovesicles secreted by cells and can be categorised into three major subtypes—exosomes, microvesicles, and apoptotic bodies—primarily according to their size range [261]. Numerous studies have demonstrated that organoid‐derived extracellular vesicles and exosomes exhibit great potential and unique advantages in signal transduction [262], repair and regeneration [263], immune response regulation [264], and biomarker detection [265], thereby attracting widespread attention.

Neural organoids contain various functional cell subpopulations, all of which secrete exosomes that play key roles in neural physiology and pathology via diverse mechanisms [266]. M2 microglia‐derived exosomes alleviate ischemic brain injury and promote neuronal survival by delivering miR‐124, which regulates the PI3K/AKT pathway to inhibit neuronal apoptosis [267, 268]. Exosomes secreted by astrocytes carried microRNAs such as miR‐190b and miR‐200a‐3p, as well as fibroblast growth factor 2 [269] and heat shock protein 70. These bioactive components can synergistically inhibit neural cell apoptosis and reduce neuroinflammatory responses [270, 271, 272]. Neural stem cell‐derived exosomes were rich in specific microRNAs, which inhibit neuroinflammation [273] and exert neurogenic/neurotrophic effects by regulating neural differentiation and secreting neurotrophic factors [274]. Thus, investigating the effects of EVs and exosomes from neural organoids on neural function merits further exploration.

#### 3.2.2 Neural Organoid–Derived EVs/Exosomes for AD and PD

Early studies have demonstrated that stem cell‐derived EVs and exosomes can restore synaptic activity in the brain, reduce cerebral inflammatory responses, enhance neural network connectivity, and modulate metabolism, highlighting their considerable potential in treating CNS diseases [275]. Inflammation is widely regarded as a key pathogenic mechanism in neurological disorders. Brain organoid‐derived exosomes contained high abundances of neurotrophin‐4 and glial cell line‐derived neurotrophic factor, which could reduce excessive reactive oxygen species production, lipid peroxidation, mitochondrial dysfunction, and the expression of pro‐apoptotic genes [259]. Additionally, lncRNA IFNG‐AS1 in exosomes derived from adipose mesenchymal stem cells acted as a molecular sponge and facilitated neurogenesis via the miR‐21a‐3p/PI3K/AKT signalling pathway, which could alleviate neuronal loss in PD and AD through functional recovery, inhibition of neuronal apoptosis, and promotion of neurogenesis [276].

The choroid plexus is a major source of cerebrospinal fluid‐derived exosomes [277]. Exosomes from choroid plexus organoids, which contained cerebrospinal fluid‐derived exosomal components, can reverse neurodegeneration in AD or ischemic brain injury by releasing brain‐specific proteins, choroid plexus‐associated proteins, and neuroprotective factors [53]. Furthermore, analysing EVs secreted by human 3D cortical organoid models from healthy controls and patients, through the use of small RNA sequencing, proteomics, and nanoparticle tracking analysis, has deepened the understanding of autism spectrum disorder, laying the groundwork for exploring EVs as biomarkers or therapeutic agents for it [260]. These findings collectively demonstrate the therapeutic potential of brain organoid‐derived exosomes in neurological diseases. Exosomes, combined with regenerative medicine, have opened new avenues for disease treatment. Moving forward, advancements in informatisation and multi‐omics integration are expected to further optimise therapeutic outcomes for CNS diseases.

#### 3.2.3 Neural Organoid–Derived EVs/Exosomes for Retinal Degeneration

Retinal degenerative diseases refer to conditions characterised by impaired or lost vision, and even severe permanent visual loss, caused by retinal dysfunction resulting from trauma or disease [278]. These diseases affect the health and quality of life of millions of people worldwide. Pathologically, the loss of retinal pigment epithelial cells or retinal neurons is a core feature of retinal degenerative diseases [21]. Similar to other parts of the CNS, the retina lacks any significant regenerative capacity. Currently, surgical and pharmacological interventions can only help slow the progression of the disease. And in most cases, they are unable to prevent disease advancement, which often leads to severe and permanent visual loss in patients [279]. Thus, there is an urgent need for novel therapeutic approaches, and researchers are investigating several alternative strategies.

Among these strategies, exosome‐based therapy is considered a promising treatment modality. Exosomes have been extensively studied in relation to various ocular diseases. They could promote the survival of retinal ganglion cells while preserving their function and reducing the expression of pro‐inflammatory cytokines. This suggested that they play a role in the repair and regeneration of retinal damage [280]. In contrast, organoid‐derived exosomes originate from organoids that were highly similar to the target tissue. Characterised by abundant bioactive molecules, good biocompatibility, and low immunogenicity, they hold great potential for the treatment of ocular diseases [281].

HiPSC‐derived 3D retinal organoids could continuously secrete extracellular exosomes. These exosomes had an average size of 100–250 nm and contained a variety of RNAs, such as hsa‐miR‐4488, which actively regulated genes involved in retinal homeostasis and developmental processes [254]. Furthermore, extracellular vesicles derived from human retinal progenitor cells were enriched in proteins related to immune regulation and retinal development, such as rhodopsin, retinal pigment epithelial cell‐specific protein, and photoreceptor cell signal transduction‐related proteins. These proteins could eliminate lipid deposition, inhibit lipotoxicity and oxidative stress, and protect RPE cells from damage caused by lipid overload [255]. Extracellular vesicles from organoid‐derived human retinal progenitor cells were internalised by retinal pigment epithelial cells and integrated into mitochondrial networks, protecting against lipid overload‐induced injury via upregulating mitochondrial fatty acid β‐oxidation‐related proteins to enhance lipid metabolism and eliminate lipid overload [108].

In experimental studies, the injection of exosome solutions extracted from retinal organoids into the eyes of Royal College of Surgeons rats resulted in reduced photoreceptor apoptosis, prevention of outer nuclear layer thinning, and preservation of visual function. In exosome solutions, hsa‐miR‐125a‐5p and hsa‐miR‐146a‐5p synergistically targeted genes associated with the MAPK pathway [258]. Similarly, in a mouse corneal epithelial wound model, exosomes extracted from hiPSC‐derived retinal organoids exerted a therapeutic effect on corneal epithelial wound healing. Compared with the control group, the exosome‐treated group exhibited significantly accelerated wound healing and increased cell proliferation, as well as reduced inflammatory markers and regulated activity of relevant signalling pathways [257].

In addition to direct exosome therapy, researchers had explored the combination of exosomes and drug treatment. For example, the hydrophobic compound cannabidiol was encapsulated in retinal organoid‐derived EVs to potentially treat age‐related macular degeneration. The results showed that cannabidiol‐loaded EVs significantly improved the survival rate of human RPE cells, reduced reactive oxygen species production, and inhibited the expression of proteins related to inflammation and apoptosis [256].

Intact organoids, cell transplantation, and exosome‐based therapy exhibit distinct advantages and applicable scenarios in neural repair [260]. Transplantation of intact organoids offers a physiological three‐dimensional structure, enabling direct tissue replacement, circuit integration, and long‐term functional reconstruction, making it suitable for large tissue defects such as stroke cavities and TBI [282]. Cell transplantation mainly provides active paracrine support and directional differentiation potential, which is advantageous for repairing focal lesions and promoting local regeneration [283]. By contrast, EVs and exosomes avoid risks such as immune rejection, tumorigenesis, and poor survival of implanted cells or organoids. As cell‐free therapeutics, they possess high stability, strong penetrability, and controllable biological activity, rendering them ideal for targeted neuroprotection and anti‐inflammatory intervention [253].

## 4 Future Directions

### 4.1 Challenges

During the translation of neural organoids toward clinical transplantation and regenerative applications, they still face multiple core bottlenecks that urgently need to be overcome [284]. For instance, neural organoids suffer from inadequate vascularisation, lack stable standardised, and reproducible production protocols for now, and are plagued by issues like post‐transplant immune rejection as well as ethical concerns related to transplantation. These challenges are key directions that need to be focused on addressing in future basic research and clinical translation.

#### 4.1.1 The Establishment of Clinical Application Standards

For future clinical applications of neural organoids, establishing uniform, clear evaluation criteria is a core prerequisite to link basic research and clinical translation [285]. Currently, the lack of standardised benchmarks causes inconsistent outcomes, hindering cross‐study comparisons and clinical validation. These criteria should cover efficacy, such as post‐transplant survival rate, neural circuit integration, CNS function improvements, and safety, such as immune rejection, abnormal proliferation, tumorigenic risks [286]. They will guide research towards clinically relevant directions, reduce blind exploration, enhance study rigour, and lay a solid foundation for repairing damaged CNS tissues and functional regeneration in neurological patients. In future clinical research, it is also important to obtain the informed consent of cell donors for organoid generation and to establish and improve a continuous global regulatory framework [287].

#### 4.1.2 Immune Rejection and Safety‐Related Issues

The primary concerns in organoid transplantation revolve around immune rejection of the graft and safety‐related issues [288]. To date, in research studies, transplantation is commonly performed using either immunodeficient animals or with the administration of appropriate immunosuppressive agents. These approaches are intended to mitigate the host's immune response against the transplanted organoids and improve graft survival. Most existing studies are limited to short‐term observations (usually ≤ 6 months) with no tumour formation detected in animal models. However, current long‐term safety data remain substantially limited, and potential risks from prolonged transplantation cannot be fully ruled out [289]. As stem cell‐derived three‐dimensional structures, organoids exhibit high cellular heterogeneity, and residual undifferentiated cells may undergo abnormal proliferation in vivo over time, leading to overgrowth or even teratoma formation. Such risks are latent and undetectable in short‐term studies. Moreover, long‐term immunosuppression may further mask or exacerbate aberrant proliferation. Therefore, long‐term tumorigenic risks cannot be dismissed solely on the basis of short‐term safety observations [290]. Moreover, some genetic modifications of iPSCs, even if intended for functional optimisation, may inadvertently disrupt normal cell growth regulation and increase long‐term tumorigenic risks [291].

#### 4.1.3 Inadequate Vascularisation

Under normal physiological conditions, the nervous system and vascular system of the human brain grow in a coordinated manner. In the culture and transplantation of CNS organoids, the lack of vascular system formation leads to slow transport of oxygen and nutrients, as well as insufficient removal of metabolic waste—these limitations restrict the growth and functional improvement of brain organoids [292]. Concurrently, this deficiency increases the expression of genes associated with hypoxia and cell apoptosis and activates metabolic stress pathways, exerting adverse effects on neural development and migration [293].

Vascularisation of brain organoids is regarded as a critical step in the research of neurological diseases [145]. The addition of signalling molecules or exogenous endothelial cells can accelerate neural differentiation [294], reduce cell death, and enhance the spontaneous electrical activity of cerebral organoids [295]. Co‐culturing CNS organoids with vascular organoids [296] or their derived vascular endothelial cells [297] enables the formation of blood vessel‐like structures within CNS organoids. Furthermore, integrating bioengineering technologies, microfluidic systems utilise precise spatial and temporal control to promote organoid vascularisation and enhance their maturation [298]. However, the full potential of these approaches has not yet been fully realised, and such innovations remain in the early stages.

The microvascular network formed in vitro by pre‐vascularisation can rapidly anastomose with the host circulation within hours to 1 day [299], significantly reducing the risk of graft central necrosis, alleviating ischemia–reperfusion injury, and improving early cell survival rate [300]. However, if the pre‐constructed blood vessels fail to stably integrate with the host system, they will degenerate, making it difficult to maintain long‐term effective perfusion, and may also induce complications such as local inflammation, thrombosis, and immune rejection [301]. In contrast, grafts relying on host in situ vascularisation can form a structurally stable and physiologically mature vascular network [302]. Nevertheless, the process of host microvascular ingrowth usually takes several days, which easily leads to central necrosis in large‐thickness grafts [303], making it more suitable for thin and low‐metabolism tissues.

In the future, combining diverse bioengineering strategies, such as vascularised organoids and 3D bioprinting technology, will effectively help improve the vascularisation of brain organoids [144, 292], generating organoids with enhanced stability, longevity, and functionality. Additionally, the introduction of high‐throughput technologies and novel biomaterials to achieve reproducibility and stability of vascularised CNS organoids may represent a direction for future research.

#### 4.1.4 Ethical Framework

The development of CNS organoids and advancements in technology have unlocked boundless possibilities for human brain research. Researchers utilise CNS organoids to investigate brain development and explore transplantation‐based recovery from brain injuries. As the complexity of brain organoids continues to advance, concerns have emerged regarding the need for additional ethical and regulatory frameworks to guide brain organoid research. Debates primarily centre on two key issues: potential “humanisation” of host animals and the question of whether brain organoids possess “consciousness” [304]. Transplanting these human “mini‐brains” into animal brains has sparked debates and unease surrounding the concept of “human‐animal brain chimeras” [305]. Currently, however, existing transplantation studies do not substantiate these concerns [305]. Most studies select the motor or sensory cortex as the transplantation target, which minimises impacts on higher brain functions. Furthermore, when transplantation is performed in brain regions associated with learning and cognition, no abnormal behaviours have been observed in experimental animals post‐transplantation [197].

Nevertheless, as research progresses, brain organoids may develop more complex, physiologically relevant structures and functions in the future. This implies that brain organoids could potentially acquire consciousness—or even higher cognitive abilities—at some point. Consequently, there is an urgent need for an ethical framework specific to brain organoid research to impose appropriate constraints on such studies.

### 4.2 Technological Optimisation and Innovation

**FIGURE 6:**
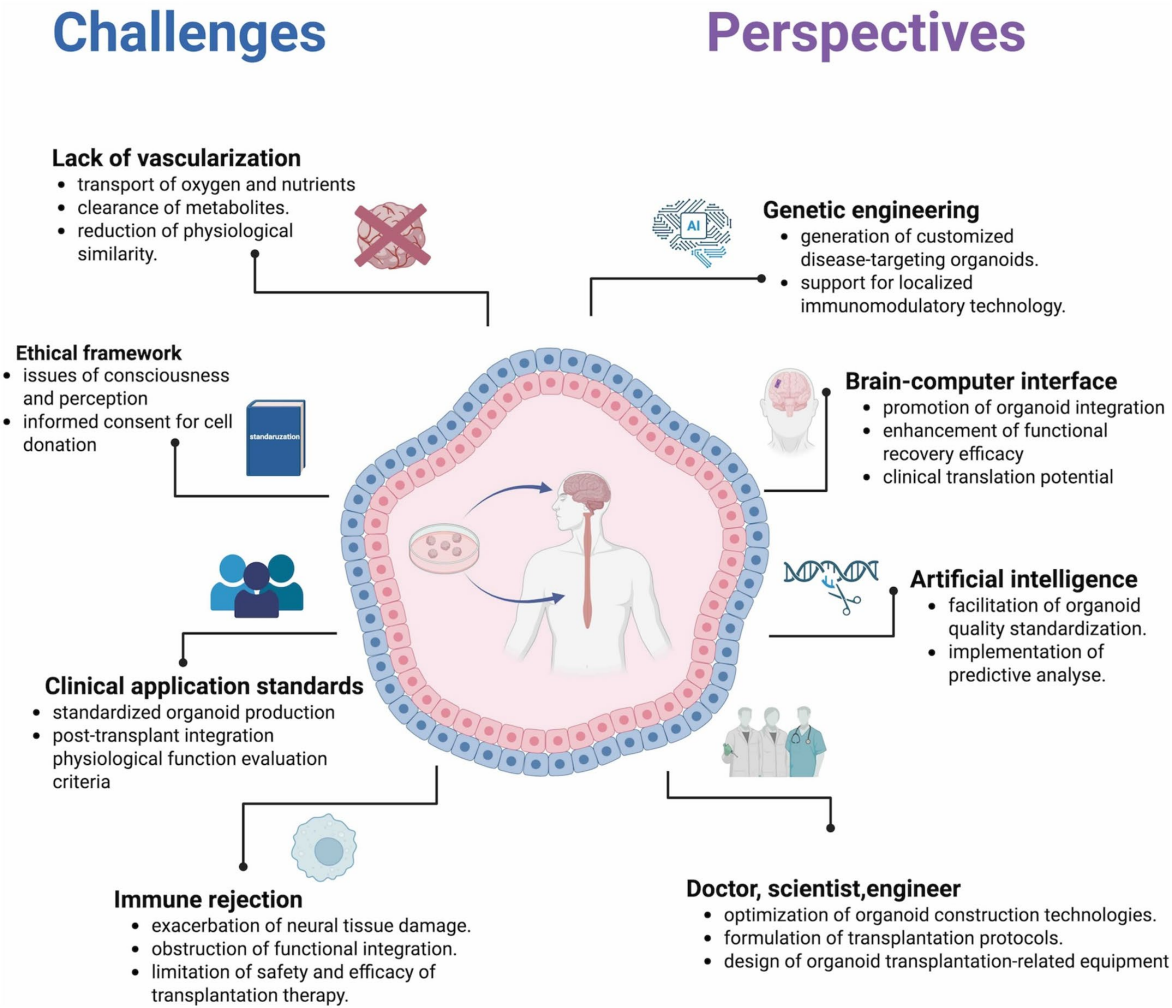
Challenges and future perspectives of neural organoids.

With the advancement of science and technology, a host of emerging technologies have emerged. Technological optimisation and innovation can address the existing shortcomings in current transplantation practices and narrow the gap between organoid transplantation research and clinical application. We propose that future optimisation and innovation efforts should focus on the following key directions, including the development of brain‐computer interfaces (BCI), advancements in genetic engineering, and the integrated application of artificial intelligence (AI) in transplantation (Figure 6).

#### 4.2.1 Integration and Application With BCI

BCI refers to a direct communication and control pathway established between the brain of a human or animal and external devices. It can bypass the peripheral nervous system and muscular system, directly capture brain electrical signals, convert these signals into device commands, and thereby realise information interaction and functional integration between the brain and the devices [306, 307]. Transplantation of brain organoids can promote neural proliferation, differentiation, maturation, axonal growth, and functional integration into the host neuronal circuitry and has been applied in a variety of applications [308]. BCIs and organoid transplantation represent two distinct strategies for restoring neural function. BCIs can enhance neural plasticity and cognitive abilities, mitigate damage, enable more interactive and rapid neural recovery [309], reestablish connectivity between the spinal cord and brain following SCI [307], and improve lower limb motor function [310].

However, their effectiveness is limited when large cavitary lesions are present. In contrast, 3D brain organoids can address these limitations and serve as an ideal alternative for transplantation [311]. The integration of BCI with organoid technology to construct innovative organoid‐brain‐computer interfaces (OBCI) holds great promise for repairing CNS injuries [312]. Electrical stimulation at the transplantation site can promote the differentiation or maturation of NSCs [313], modulate neural tissue plasticity, guide axonal growth direction, reduce lesion volume, enhance organoid connectivity with brain structure and function [163], and improve post‐injury sensory‐motor and cognitive deficits [314]. OBCIs enhance the structural and functional connectivity between grafts and the host brain, promote the host's functional recovery, and have demonstrated safety and feasibility. Specifically, OBCIs repair damaged brain tissue through regeneration and regulatory mechanisms, thereby restoring the normal function of neural networks [315].

Future research aims to identify innovative materials suitable for brain‐computer interfaces to improve treatments for neurological disorders. Implantable hydrogels, with their excellent biocompatibility and electrochemical properties, effectively record neural signals and enable neuromodulation, offering broad prospects for application in brain‐implanted electrodes [316]. Carbon nanomaterials can enhance interface stability, flexibility, and efficacy, potentially laying the foundation for developing next‐generation high‐performance BCIs with enhanced functionality and longevity [317].

#### 4.2.2 Integration and Application With Gene Therapy

Integrating genetic engineering approaches enables the precise regulation of cell differentiation direction and functional characteristics in neural organoids, thereby generating customised organoids with disease‐targeting properties or host compatibility. This strategy not only enhances the matching degree between organoids and the patient's pathological microenvironment but also provides core technical support for personalised transplant therapy, drug screening, and mechanism research of neurological diseases, further advancing the implementation of personalised medicine.

To date, genetically modified organoids have been utilised across a broad spectrum of biomedical fields, including cancer research and therapy, the establishment of in vitro disease diagnostic models, preclinical drug screening, gene correction, organ development studies, and tissue repair and regeneration via diverse mechanisms. A key strategy is the genetic modification of organoids using gene‐editing technologies, such as the CRISPR‐Cas9 system, to improve their therapeutic effectiveness. For instance, the combination of brain organoids with CRISPR/Cas9 enables researchers to introduce or correct mutations associated with AD and investigate their effects in the context of 3D human cell models, facilitating more precise exploration of the disease mechanisms underlying AD [318].

In retinal organoids derived from patients with Leber congenital amaurosis type 7 harbouring *CRX* mutations, allele‐specific CRISPR/Cas9‐mediated gene editing was employed to knock out the mutant *CRX* allele. This intervention rescued photoreceptor defects in these retinal organoids, demonstrating potential for targeted treatment of inherited retinal degenerations [319]. In another application, thymic organoids were engineered to alleviate the impaired differentiation potential caused by *RAG2* deficiency, offering therapeutic prospects for severe combined immunodeficiency [320]. Additionally, genome‐edited *Islet1*−/− retinal organoids have been shown to further improve host‐graft synaptic connectivity and visual function recovery following transplantation [251]. Beyond these, genetic modification also supports the development of localised immunomodulatory technologies for cell and organoid transplantation, addressing critical immune compatibility challenges [321].

#### 4.2.3 Integration and Application With Artificial Intelligence

AI can analyse large and complex datasets, enabling a comprehensive evaluation of multiple factors [322]. This provides a revolutionary approach for medical research exploration. In the context of organ transplantation, an AI application optimises donor‐recipient matching and enhances predictive capabilities for organ transplantation outcomes [323]. Beyond organ transplantation, AI‐driven machine learning techniques contribute to optimising processes in tissue engineering and 3D bioprinting, as well as refining the design of organoids. The establishment of robust AI models allows for the extraction of critical information from organoid characteristics [324], efficient processing of multi‐omics data, and implementation of predictive analyses, holding promise for achieving controllable quality and standardisation of organoids [325]. Additionally, AI facilitates the computation and visualisation of morphological and fluorescent features during organoid development [326]. Notably, it enables the successful identification and prediction of retinal differentiation in organoids before the onset of reporter gene expression [327] and supports the identification of genuine biomarkers for brain diseases [328].

AI also enables rapid and accurate analysis of organoid structure and function, with its predictive capabilities directly serving the design of tissue repair strategies. Furthermore, AI‐powered automated imaging analysis provides critical technical support for researching the long‐term stability of organoids in regenerative medicine.

The integration of AI and regenerative medicine exhibits tremendous transformative potential, offering prospects for advancing effective therapies and innovative treatment strategies. In the future, with the assistance of AI, it is expected to achieve the standardised in vitro generation of characterised CNS organoids—organoids that can be applied to the regenerative repair of CNS injuries.

## 5 Conclusion

The development of neural organoids offer new hope for the repair and regeneration of the CNS. Emerging technologies such as bioreactors, 3D printing, microfluidic systems, and bioelectronic interfaces can facilitate the efficient generation of neural organoids. However, the clinical translation of neural organoids is still hampered by key bottlenecks: insufficient maturity and vascularisation of the organoids themselves, lack of standardised protocols in production, and difficulty in achieving effective functional integration with the host after transplantation. In the future, interdisciplinary collaboration among basic researchers, engineers, and clinicians is required, along with further integration of cutting‐edge technologies like brain‐computer interfaces, genetic engineering, and AI. This will provide more promising strategies to overcome existing impediments and advance regenerative medicine.

## Author Contributions

Y.W. and J.L. wrote the manuscript. J.L. and X.M. prepared figures and tables. W.W., X.H., and Y.L. searched and checked the literature and revised the manuscript. J.Z., F.Z., and L.W. revised the manuscript, supervised the research, and revised the manuscript. L.W. provided financial support.

## Funding

This work was supported by the National Natural Science Foundation of China (82172527) and Natural Science Foundation of Zhejiang Province (LZ26H090001).

## Conflicts of Interest

The authors declare no conflicts of interest.

## Data Availability

Data sharing not applicable to this article as no datasets were generated or analysed during the current study.
